# Association of Paternal Diabetes With Hemorrhagic Stroke in Bangladeshi Women: The MAGPIE Study

**DOI:** 10.1002/hsr2.70809

**Published:** 2025-04-29

**Authors:** Md. Abdullah Yusuf, Redoy Ranjan, Dipannita Adhikary, Adneen Moureen, Maliha Hakim

**Affiliations:** ^1^ Department of Microbiology National Institute of Neurosciences and Hospital Dhaka Bangladesh; ^2^ Department of Biological Sciences Royal Holloway University of London London UK; ^3^ Department of Cardiac Surgery Bangabandhu Sheikh Mujib Medical University Dhaka Bangladesh; ^4^ Tuberculosis New Technologies and Diagnostics, USAID's Dhaka Bangladesh; ^5^ Department of Neurology National Institute of Neurosciences and Hospital Dhaka Bangladesh

**Keywords:** Bangladeshi, hemorrhagic stroke, paternal diabetes, women

## Abstract

**Background:**

Hemorrhagic stroke (HS) accounts for ~10% of all first‐time strokes, with an increasing incidence in Asia and a scarcity of information available about sex‐related differences. This study investigates the independent predictors of HS among Bangladeshi women.

**Methods:**

The Multidimensional Approach of Genotype and Phenotype in Stroke Etiology (MAGPIE) study is a Bangladeshi observational study that recruited nationwide HS patients between 2022 and 2024. We utilized univariate analysis to identify risk patterns in the data sets, whereas a multivariate logistic regression (LR) analysis identified the independent predictors of HS in women.

**Results:**

We evaluated a total of 1080 hemorrhagic patients with female predominance (59.5%) and similar age of onset to males (*p* = 0.38). Although males had significantly higher rates of hypertension (*p* < 0.001), diabetes (*p* = 0.008), chronic constipation (*p* = 0.03), and systolic blood pressure (*p* < 0.001), females were found to have significantly higher body mass index (23.1 ± 3.1 vs. 21.4 ± 2.9; *p* < 0.001) and paternal diabetes (9.6% vs. 4.8%; *p* = 0.004) compared to male. Further, the age‐adjusted multivariate LR model found paternal diabetes (OR 2.1, 95% CI 1.2–3.5, *p* = 0.007) as a potential independent predictor of HS in females compared to males. The area under the receiver operating characteristic (AUROC) curve of 0.67 (95% CI 0.63–0.70, *p* < 0.001) with 67.7% sensitivity and 58.2% specificity presents the goodness of fit of the model.

**Conclusion:**

Bangladeshi women with a history of paternal diabetes have a 2.1‐fold heightened risk of HS than men.

## 1 Introduction

Hemorrhagic stroke (HS) due to intracerebral hemorrhage (ICH) accounts for about 10% of first‐time strokes and presents a significant health challenge due to its high prevalence and mortality rates in Asia and globally, but there is a paucity of data on sex‐related differences [1, 2, 3]. Despite limited data, approximately 68 out of every 100,000 people experience HS annually, which constitutes about 25% of all strokes in Bangladesh [3, 4, 5]. In addition to conventional risk factors [2, 4] like hypertension (HTN), diabetes mellitus (DM), and smoking, anthropometric measurements, including body mass index (BMI), are closely linked to the risk and severity of HS [6, 7, 8]. HTN affects 55%–75% of the Bangladeshi population, which is a potential predictor of HS and further emphasizes the public health burden [9, 10, 11, 12].

Although the relationship between anthropometric measurements and stroke risk varies globally and by gender, higher BMI is linked to HTN, a major risk factor for HS [6, 13]. However, Southeast Asians with a lower BMI might have a higher risk of HS if they have higher central obesity [6, 8]. In Bangladesh, the mortality rate for HS is around 50%, primarily due to restricted access to medical care, delays in treatment, and challenges in timely stroke management [3, 5]. Region‐specific strategies, like enhancing healthcare facilities and public awareness, encouraging preventive strategies like lifestyle changes, and targeting tailored abdominal obesity, may further aid in assessing and reducing stroke risk across diverse populations [6, 10, 11, 12, 13, 14].

Familial factors, particularly paternal diabetes, may increase the risk of HS in women and can be attributed to genetic susceptibility, shared lifestyle factors, and epigenetic influences [15, 16, 17]. Women may inherit insulin resistance, vascular dysfunction, and pro‐inflammatory pathways from their diabetic fathers, making their cerebrovascular systems more vulnerable to HS, regardless of their metabolic health [18, 19, 20]. Nevertheless, determining if a father's diabetes affects his daughter's HS risk could enhance understanding of inherited vulnerabilities and improve early risk assessment and targeted screening for women at higher stroke risk [15, 21]. Addressing this research gap will enhance precision medicine approaches, leading to more personalized prevention and management of cardiovascular diseases like HS in women.

This study aimed to evaluate the association between family comorbidity, particularly stroke and diabetes, and HS due to ICH in Bangladeshi women than men.

## 2 Patients and Methods

### 2.1 The MAGPIE Study

The Multidimensional Approach of Genotype and Phenotype in Stroke Etiology (MAGPIE) is an observational study with a protocol published in detail elsewhere [22]. However, briefly, the MAGPIE study has recruited adult HS patients aged ≥ 18 years at a tertiary care center known as the National Institute of Neurosciences and Hospital (NINS&H) in Bangladesh. We collected extensive phenotypic clinical data on ICH patients across Bangladesh between 2023 and 2024. Two independent experts, a neurologist and an intervention radiologist, evaluated neuroimaging CT or MRI scan findings to confirm the diagnosis of ICH representing HS participants. The MAGPIE study included HS patients due to ICH with mild to severe conditions, as well as those with a history of prior or recurrent HS, including seriously ill or mechanically ventilated patients, to avoid selection and reporting bias. However, individuals with significant comorbidities, like neurological disorders (e.g., brain tumors) or HS from other conditions (e.g., advanced cancer, trauma) or medications affecting HS risk, were excluded from the study.

The study variables were sociodemographic variables (age, sex, HTN, DM, chronic kidney disease [CKD], smoking, hypercholesterolemia, ischemic heart disease [IHD], chronic constipation, and blood pressure measurements [both systolic and diastolic] at the time of admission and anthropometric measurements, specifically BMI). Additionally, we recorded the family history of comorbidities, specifically stroke and DM, based on self‐reports from the participants. To ensure data accuracy, we collected self‐reported information using neutral wording, standardized questionnaires, and clear instructions while encouraging honest responses using multiple response methods. The institutional review board of the NINS&H, Bangladesh, granted ethical clearance for the study (NINS/2024/358) and obtained consent from all participants or their next of kin in the case of unconscious or intubated patients. The study was conducted according to the Declaration of Helsinki.

### 2.2 Statistical Analysis

We utilized statistics software IBM SPSS version 28.0 (NY, USA) for data analysis, summarizing results with percentages and mean ± SD for categorical and continuous variables, respectively. Initially, univariate analysis assessed the risk associated with independent variables, and an age‐adjusted multivariate logistic regression (LR) model identified independent predictors of HS among women. For the LR model, we utilized all independent variables except with zero events, having a *p*‐value of < 0.05, and considered the mean value of systolic blood pressure (SBP) on admission (≥ 145 mmHg) and BMI ≥ 22.4 kg/m² as categorical variables to assess the risk of HS occurrence among women. The *p*‐value was reached from the chi‐square test and independent *t*‐test, as appropriate. The area under the receiver operating characteristic (AUROC) curve was used to evaluate the model's goodness of fit. Furthermore, the sensitivity, specificity, positive predictive value (PPV), and negative predictive value (NPV) of independent predictors validate the accuracy of the risk prediction model. Data quality assessment was performed using Little's MCAR (Missing Completely At Random) test, which assesses missing data sets [23]. We also checked for multicollinearity among independent variables using the collinearity tolerance and variance inflation factor (VIF) value to observe the significant multicollinearity that needed to be addressed [24]. Statistical significance was established with a *p*‐value threshold of < 0.05.

## 3 Results

We evaluated a total of 1080 HS patients with female predominance (*n* = 643; 59.5%) and similar age of onset between males and females (55.9 ± 14.2 vs. 55.2 ± 13.5; *p* = 0.38) (Table 1). We found that males had significantly higher HTN (78% vs. 67.9%; *p* < 0.001), diabetes (15.3% vs. 12.8%; *p* = 0.008), chronic constipation (11.7% vs. 7.9%; *p* = 0.03), smoking (36.6% vs. 0.0%, *p* < 0.001), and SBP on admission (150.8 ± 25.3 vs. 140.7 ± 21.8; *p* < 0.001) compared to female. In contrast, females had significantly higher BMI (23.1 ± 3.1 vs. 21.4 ± 2.9; *p* < 0.001) and paternal diabetes (9.6% vs. 4.8%; *p* = 0.004) than males.

**Table 1:** Baseline characteristics of study population.

Variables	Male (*N* = 437)	Female (*N* = 643)	*p*‐value
Age (mean ± SD)	55.9 ± 14.2	55.2 ± 13.5	0.38
Hypertension	341 (78.0%)	436 (67.9%)	< 0.001
Hypercholesterolemia	5 (1.1%)	5 (0.8%)	0.54
Diabetes mellitus	67 (15.3%)	82 (12.8%)	0.008
Chronic kidney disease	5 (1.1%)	4 (0.6%)	0.35
Ischemic heart disease	7 (1.6%)	6 (0.9%)	0.32
Chronic liver disease	2 (0.5%)	1 (0.2%)	0.35
Chronic constipation	51 (11.7%)	51 (7.9%)	0.03
Smoking	160 (36.6%)	0 (0%)	< 0.001
Family history of stroke	83 (19.0%)	124 (19.3%)	0.90
Maternal stroke	25 (5.7%)	56 (8.7%)	0.06
Paternal stroke	42 (9.6%)	51 (7.9%)	0.33
Sibling stroke	16 (3.7%)	24 (3.7%)	0.95
Family history of DM	100 (22.9%)	175 (27.2%)	0.10
Maternal DM	32 (7.3%)	66 (10.3%)	0.09
Paternal DM	21 (4.8%)	62 (9.6%)	0.004
Sibling DM	55 (12.6%)	67 (10.4%)	0.26
SBP on admission	150.8 ± 25.3	140.7 ± 21.8	< 0.001
DBP on admission	88.4 ± 15.4	88.2 ± 13.2	0.86
HbA1c	6.2 ± 1.3	6.1 ± 1.3	0.64
Body mass index	21.4 ± 2.9	23.1 ± 3.1	< 0.001

*Note:* Continuous variables are expressed as mean ± SD and categorical variables as percentages (%). The *p*‐value was reached from the chi‐square test and independent *t*‐test, as appropriate.

Abbreviations: DBP, diastolic blood pressure; DM, diabetes mellitus; SBP, systolic blood pressure.

**Figure 1:**
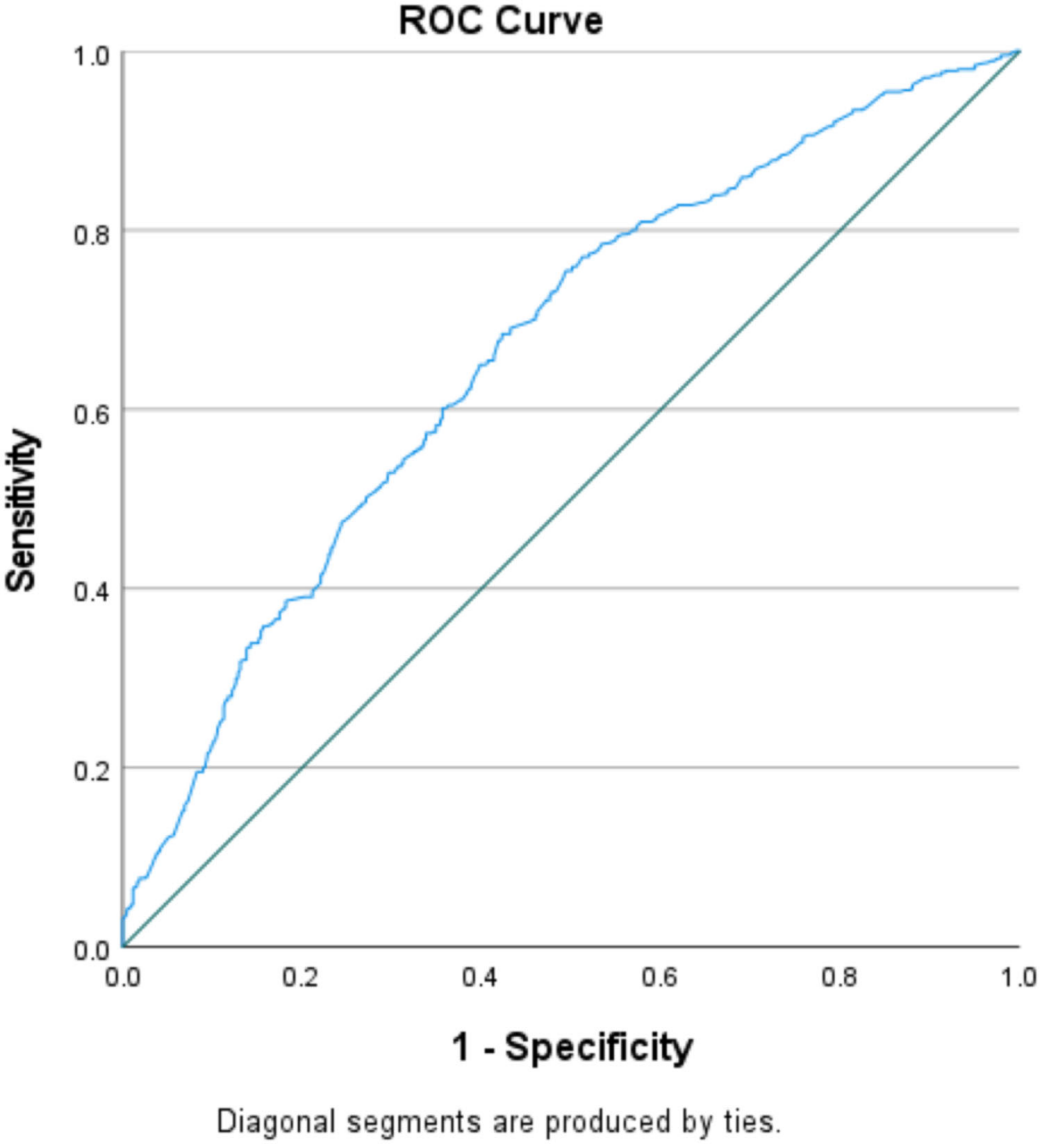
The ROC curve demonstrating goodness of fit of the risk prediction model.

Further, the age‐adjusted multivariate LR model found paternal DM (OR 2.1, 95% CI 1.2–3.5, *p* = 0.007) as a strong potential independent predictor of HS in females compared to males (Table 2). Further, the OR of 0.56 (95% CI 0.43–0.73, *p* < 0.001) among females with SBP ≥ 145 mmHg on admission indicates that women have 44% lower odds of experiencing HS than males. The AUROC curve, 0.67 (95% CI 0.63–0.70, *p* < 0.001) with 67.7% sensitivity and 58.2% specificity presents the goodness of fit of the model (Figure 1). Paternal DM has sensitivity, specificity, PPV, and NPV in predicting HS in females were 9.6%, 98.2%, 74.7%, and 41.7%, respectively. However, the Little MCAR test confirmed that the missing data were random, and multicollinearity was not significant.

**Table 2:** Age‐adjusted multivariate LR model independent predictors of hemorrhagic stroke in female.

	*p*‐value	Odds ratio	95% confidence interval	
			Lower	Upper
Age	0.14	1.0	0.99	1.01
Paternal DM	0.007	2.09	1.21	3.59
SBP ≥ 145 mmHg	< 0.001	0.56	0.43	0.73
DM	0.05	0.98	0.97	1.00
Chronic constipation	0.15	0.73	0.47	1.12
BMI ≥ 22.4 kg/m^2^	0.24	1.19	0.88	1.62

*Note:* Variable(s) entered: age, DM, chronic constipation, SBP admission ≥ 145 mmHg, BMI ≥ 22.4, and paternal DM.

## 4 Discussion

This is the first Bangladeshi study to find that paternal DM increases ~2 times the risk of HS in women than in men. Despite HTN being an established risk factor for HS, women with SBP ≥ 145 mmHg have 44% lower odds of experiencing HS compared to men.

Despite a family history of stroke, it is a known risk factor for HS in young women [25]; this is the first study to find that paternal DM is a potential predictor of increases in the risk of HS among women than men. Ylinen et al. [26] found that a history of maternal stroke increases the risk of heart stroke among Finnish individuals with Type 1 diabetes; however, parental risk factors appear to have a limited impact on stroke risk. On the contrary, Framingham study [27] found that parental stroke by 65 years of age was associated with a threefold increase in risk of offspring stroke. Although the pathophysiology is unknown, paternal DM may increase the risk of HS in women more than men due to a combination of genetic, epigenetic, and hormonal factors, which need to be confirmed by a large‐scale genetic analysis.

Women with a history of paternal DM may inherit a predisposition to insulin resistance or impaired glucose metabolism, amplifying their vulnerability to vascular complications [25, 26, 27, 28]. Furthermore, estrogen, which plays a protective role in vascular health, may be less effective in reducing damage caused by inflammation or oxidative stress related to diabetes [29, 30]. Genetic imprinting from the paternal side may also contribute to different risk patterns, which collectively increase the likelihood of endothelial dysfunction and compromise blood vessel integrity, which can lead to an increased risk of HS in women [31, 32, 33]. Additionally, existing studies [34, 35] observed that uncontrolled HTN causes ~3.5 times higher risk of HS, supporting our study findings. A recent Swedish study [36] found that ≥ 145 mmHg SBP was associated with a > 2‐fold higher risk of HS compared to 130 mmHg, whereas Kim et al. [37] found that, for each 20 mmHg rise in SBP, the risks of ICH were ~2.5 times and ~3.2 times higher in men and women, respectively. We also found a link between high SBP and the risk of HS; however, this study first described that women had lower odds of experiencing HS than men, despite having an SBP of ≥ 145 mmHg.

Like any study, it is important to recognize this study's limitations, especially its observational nature; however, using the most extensive study sample mitigates the risk of bias. Further, this study recruited Bangladeshi adult HS patients, so our study findings may not be applicable to those of other ancestries and child patients. Given that the majority of the population in Bangladesh is Muslim, we lacked detailed data on the religion of participants, as different lifestyles and dietary habits might influence the occurrence of cardiovascular diseases, such as stroke in women [38]. Although this study included referred patients from across the country, recruiting from every geographical region was impossible. Despite the novelty of a relatively lower risk of HS in women than in men with an SBP of ≥ 145 mmHg, exploring the pathophysiology was beyond the scope of this study. Although our sample is sizable, a short recruitment period and recruiting only from a tertiary care hospital in the capital of Bangladesh can lead to selection bias, as participants primarily consist of patients with more severe conditions, limiting diversity and excluding those treated in primary healthcare settings. Although the low sensitivity of paternal diabetes in predicting women's HS is a concern, the good specificity and PPV in a small sample of paternal diabetes help clarify these concerns and mitigate the risk of bias [39, 40]. Although participants' self‐reported paternal diabetes may introduce bias, previous studies found that self‐reporting data are generally reliable by ensuring anonymity, consistency, and a standardized questionnaire [41, 42]. Further, we lack data on the details of the brain lesion (e.g., size of the lesion and midline shift) and treatment outcomes, which prevents us from assessing differences in mortality or morbidity in the study population across genders.

## 5 Conclusion

Bangladeshi women with a paternal history of DM have a 2.1‐fold increased risk of HS compared to men. We recommend conducting comprehensive genetic studies to validate these findings and early optimization of associated factors to reduce the risk of HS in this population.

## Author Contributions

**Md. Abdullah Yusuf:** conceptualization, investigation, methodology, validation, visualization, data curation, writing – review and editing, resources, supervision. **Redoy Ranjan:** conceptualization, investigation, methodology, validation, visualization, writing – review and editing, writing – original draft, formal analysis, resources. **Dipannita Adhikary:** conceptualization; methodology, validation, formal analysis, resources, writing – review and editing, visualization. **Adneen Moureen:** conceptualization, methodology, validation, supervision, resources, writing – review and editing, visualization. **Maliha Hakim:** conceptualization, methodology, validation, supervision, resources, writing – review and editing, investigation.

## Conflicts of Interest

Redoy Ranjan is an Editorial Board member of *Health Science Reports* but was excluded from all editorial decision‐making related to the acceptance of this article for publication. The other authors declare no conflict of interest in the publication of this paper.

## Transparency Statement

The lead author Md. Abdullah Yusuf affirms that this manuscript is an honest, accurate, and transparent account of the study being reported; that no important aspects of the study have been omitted; and that any discrepancies from the study as planned (and, if relevant, registered) have been explained.

## Data Availability

The data supporting this study's findings are available upon request from the corresponding author.
